# Editorial of Special Issue “The Interplay of Microbiome and Immune Response in Health and Diseases”

**DOI:** 10.3390/ijms20153708

**Published:** 2019-07-29

**Authors:** Amedeo Amedei, Gwendolyn Barceló-Coblijn

**Affiliations:** 1 Department of Experimental and Clinical Medicine, University of Florence, 50134 Florence, Italy; 2 SOD of Interdisciplinary Internal Medicine, Azienda Ospedaliera Universitaria Careggi (AOUC), 50134 Florence, Italy; 3 Lipids in Human Pathology, Health Research Institute of the Balearic Islands (IdISBa, Institut d’Investigació Sanitària Illes Balears), 07120 Palma, Balearic Islands, Spain

Increasing data suggests and supports the idea that the gut microbiota (GM) modulates different host pathways, playing a crucial role in human physiology and consequently impacting in the development of some pathologic conditions. Explorations of how the microscopic communities might contribute to health or disease have moved from obscure to ubiquitous. Recently, studies have linked our microbial settlers to inflammatory bowel diseases (IBD)s, obesity, asthma, autism spectrum disorders, stroke, diabetes, and cancer. In agreement with Hanage, who suggested a scepticism dose about the predominant role of microbiota [1], we have edited this special issue with the aim to publish manuscripts respecting this spirit of scientific rigor to the detriment of enthusiasm (which often characterizes GM studies).

However, there is no doubt that microbial metabolites bridge various, even distant, areas of the organism by way of the hormone and immune system, contributing to the development of different pathologies, such as the autoimmune disorders, as discussed by Gianchecci et al. [2]. The impact of a GM imbalanced in autoimmunity pathogenesis has been suggested by different experimental evidence, and physiological mechanisms, (i.e., the establishment of immune homeostasis) are influenced by commensal bacteria. Microbiota alterations generate effects in the immune system, such as intestinal inflammation, enhanced gut permeability, and defective tolerance to food antigens. In particular, early findings reported differences in the gut microbiome of subjects affected by several autoimmune conditions, including prediabetes. 

In addition, the microbiota seen also have implications in the therapeutic approaches of lymphoid malignancies and immunotherapy-based cancer treatments. Zuccaro et al. [3], discussed the microbiota impact during chemo-free treatment of lymphoid malignancies. To date, no studies have been planned to evaluate the GM composition in patients with lymphoproliferative disorders (and treated with chemo-free therapies), and the probable association between GM, treatment outcome, and immune-related adverse events has never been analysed. The authors remark the necessity of additional studies to make opportunities for a more personalized approach in the patients’ subset. 

During the last few years, the GM has gained increasing attention as a consequence of its immunomodulator role. In particular, with the introduction of checkpoint inhibitors’ immunotherapy and adoptive cell transfer in oncology, these findings became of primary relevance in light of experimental data that suggested microbiota involvement as a credible predictor of responsiveness. These impacting themes have been discussed by Brandi et al. [4], who reviewed the GM implication in anti-cancer immunotherapy strategies, remarking the need to identify the specific GM actions and develop innovative strategies to favourably edit its composition. 

It is important to link microbiota alterations (dysbiosis) and intestinal-correlated diseases. In this regard, the manuscript of Lopetuso et al. [5] is interesting, since it explores the role of bacteriocins and bacteriophages in the most recurrent gastrointestinal disorders, speculating on their potential therapeutic application. The bacteriocins are bactericidal peptides (produced by both gram+ and gram- bacteria) with an inhibitory activity against diverse groups of undesirable microorganisms. Conversely, the bacteriophages are viruses that are able to infect bacteria, forcing them to produce viral components. Bacteriocins and bacteriophages can influence both human health and diseases because they modulate the intestinal microbiota and regulate the relationships between different microorganisms, strains, and cells living in the human gut. 

However, one of the most important messages that this special issue conveys is that we are still far from understanding the full extent of GM actions on human health and the impact of its manipulation. Cianci et al. [6] systematically reviewed these advances, linking gut microbiota not only to colorectal cancer, but also to oesophageal, stomach, and pancreatic cancer, and hepatocellular carcinoma. Hence, the GM action appears to go beyond the direct effect on the intestines, reaching those districts that may not be directly colonized by the various microbial species. This is crucially important when designing new therapies, including surgery and radiotherapy, aiming to restore the damaged microbiome during assessment of their impact on a patient’s health. This concept is extensively covered by Toor et al. [7] in which the authors stressed their concerns on the impact of the microbiome on the uncontrolled use of antibiotics (which is also a current major concern for the Public Health Authorities), chemotherapeutic drugs, or even changes in dietary patterns. The authors not only summarized state-of-the-art strategies to study gut microbiomes, but they also included new strategies to manage dysbiosis through diet, bile acids, and immune pharmaceutics. 

It is clear that one of the major concerns in the field is how immunotherapy may affect the delicate equilibrium existing in the microbiome ecosystem, and vice versa. Picchianti–Diamanti et al. [8] addressed this question in the context of rheumatoid arthritis (RA). In a pilot study, the authors demonstrated that in addition to oral microbiota dysbiosis, gut dysbiosis was also detected. Hence, the comparison of the impact of intestinal microbiota in three groups of RA patients and patients receiving methotrexate and/or etanercept (a biotechnological agent targeting TNF-alpha) led the authors to conclude that part of the benefits of this treatment is related to the partial restoration of the beneficial microbiota. 

However, the scenario gets more complex when considering the connections established by distant organs, such as gut-associated lymphoid tissue (GALT, explained by Toor et al. [7]) or the gut-liver axis reviewed by Milosevic et al. [9]. Milosevic et al. evaluated another GM aspect, the so called “gut-liver axis”, which has attracted great attention in recent years. GM communication is bi-directional and involves endocrine and immunological mechanisms. In this way, gut-dysbiosis and composition of “ancient” microbiota could be linked to the pathogenesis of numerous chronic liver diseases, such as chronic hepatitis B and C, alcoholic liver disease, development of liver cirrhosis, and finally the hepatocellular carcinoma. The authors discussed the current evidence supporting a GM role in the management of these different chronic liver diseases and potential novel therapeutic GM targets, such as fecal microbiota transplants, antibiotics, and probiotics. 

Detecting the microbial interactions is essential to understand the GM structure and function. In a mouse model, Liu et al. [10] inferred the microbial co-occurrence patterns using a random matrix theory-based approach in the GM in response to chondroitin sulfate disaccharide (CSD) under healthy and stressed conditions. A total of 34 operational taxonomic units (OTU) were identified as module hubs and connectors, likely acting as generalists in the microbial community. In particular, *Mucispirillum schaedleri* acted as a connector in the stressed network in response to the CSD supplement and may play a crucial role in bridging intimate interactions between the host and its microbiome. In addition, several modules correlated with physiological parameters were detected. A positive correlation between node connectivity of the proteobacteria with superoxide dismutase activities under stress suggested that proteobacteria can be developed as a potential pathological marker. These results provided novel insights into GM interactions and may facilitate future endeavours in microbial community engineering, directly influencing some molecular pathways. 

The GM role is being extensively studied in the context of chronic inflammatory diseases, in particular in inflammatory bowel diseases (IBDs), which have a multifactorial etiology (not firmly established yet). The fact that IBD incidence is steadily increasing in developed and developing countries clearly suggests that lifestyle changes are key players in the onset of these diseases. Many studies have established that the GM biodiversity is frequently altered in IBD patients, in particular because of the reduction in firmicutes and an increase in proteobacteria. In this situation, IBD patients are highly vulnerable to any opportunistic pathogen, such as *Candida (C) albicans*, a serious clinical problem due to the high associated morbidity and mortality. Consequently, the *C. albicans* infection complicates the IBD treatment, as the anti-inflammatory compounds most commonly prescribed do not have antifungal activity. With the aim to identify new compounds showing this dual effect, i.e., compound having simultaneously antifungal and anti-inflammatory properties, Bortolus et al. [11] investigated the antifungal properties of a novel compound, 2,3-dihydroxy-4-methoxybenzaldehyde (DHMB). Using in vitro and in vivo models (murine DSS-induced colitis model), the authors demonstrated the great potential this aromatic molecule has as an antifungal agent with anti-inflammatory properties. On the other hand, Charlet et al. [12] investigated an alternative approach widely used in the management of various inflammatory and autoimmune diseases: Immunotherapy with intravenous immunoglobulin (IVIg). Using the same murine model, the authors demonstrate that this treatment has a clear impact on GM composition, decreasing the content in *Escherichia coli*, *Enterococcus faecalis*, and *C. albicans* populations. Conversely, the beneficial effects of IVIg were associated with the suppression of inflammatory cytokine IL-6 and the enhancement of IL-10 and PPAR-gamma (involved in inflammation resolution). Hence, it seems that the beneficial effects of IVIg in infectious diseases goes beyond a simple neutralization of microbes, acting actively on anti-inflammatory pathways, which turned out to be critical for protection against infection.

Importantly, all the basic concepts and general approaches developed while studying gut microbiota may also apply, to a greater or lesser degree, to other biological systems. such as the vaginal or skin ecosystems. This special issue contains a comprehensive review by Torcia [13] on the interplay among vaginal microbiome, immune response, and sexually transmitted infections (STIs). In addition to the role that the cervico-vaginal microbiota has during egg fertilization and pregnancy, its maintenance is key in the prevention of infectious pathogens, particularly during the transmission of the human immunodeficiency virus (HIV), the human papilloma virus (HPV), and the herpes simplex virus 2 (HSV2). Furthermore, an increased risk of STI acquisition is clearly associated to vaginal dysbiosis. Torcia [13] described the current knowledge on how the immune system, epithelial cells, and microbiota are interconnected and discussed different prevention strategies. The latter has become a worldwide health issue due to the high incidence of STIs in low- and middle-income countries and due to their resurgence in developing countries. 

Finally, Park et al. [14] discussed the GM role in the largest organ in the human body: The skin. As in the gut, liver, or vagina, the pathological alteration of the microbiome system often leads to inflammation. Interestingly, the authors described the opposite impact on the skin health of two members of the same genus, *Staphylococcus (S) aureus* and *S. epidermidis* and they warn about the importance of understanding how these two species can modulate the cutaneous-immune response prior to manipulating their levels as part of a treatment. It is clear that this warning should be issued for any microbiome ecosystem. 

In other words, the different studies and data presented and discussed in this special issue suggest the microbiota centrality in the development and maintenance of the “health” and in favouring (those cases in which the microbiota’s complex relational architecture is dysregulated) the onset of pathological conditions. The intricate relationships between the microbiota and human beings, which invest core notions of biomedicine, such as “health” and “the individual,” concern not only problems of an empirical nature, but seem to require the need to adopt new concepts and novel perspectives in order to be properly analysed and utilized, especially for their therapeutic implementation. In this context, it is very adequate the contribution of Amedei et al. [15], which illuminates the discussion of the theoretical proposals and innovations (from the ecological component to the notion of the polygenomic organism) aimed at producing this perspective change. In conclusion, the authors analysed what impact and what new challenges these novel approaches might have on personalized medicine. 
